# Traumatic lateral atlantoaxial dislocation combined with a type II odontoid fracture in a patient with ankylosing spondylitis: A case report

**DOI:** 10.1097/MD.0000000000030912

**Published:** 2022-10-07

**Authors:** Jongpil Eun, Youngmin Oh

**Affiliations:** a Department of Neurosurgery, Research Institute of Clinical Medicine of Jeonbuk National University, Jeonbuk National University Medical School and Hospital, Jeonju, Korea.

**Keywords:** ankylosing spondylitis, atlantoaxial dislocation, atlantoaxial instability, neck pain, odontoid fracture, trauma

## Abstract

Traumatic lateral atlantoaxial dislocation (AAD) combined with an odontoid fracture is extremely rare. The standards applicable to management of the traumatic lateral AAD are still in flux due to the infrequency of this injury.

**Patient concerns:**

We present a unique case of traumatic lateral AAD combined with a type II odontoid fracture in a patient with ankylosing spondylitis (AS).

**Diagnosis:**

Spinal computed tomography showed ankylosis of the entire spine from the sacroiliac joint to the cervical spine. On the cervical X-ray the head was rotated to the right with anterior subluxation of the C1 and odontoid tip relative to C2. The coronal computed tomography (CT) scan also revealed left lateral dislocation of C1 on C2 with a horizontal translation of the odontoid tip. On the axial and sagittal CT scan, the left C1 lateral mass was displaced anteriorly and locked by C2 body

**Interventions:**

We performed occipito-cervical fusion (OCF) after successful manual reduction under general anesthesia.

**Outcomes:**

The patient’s recovery from surgery was uneventful and without complication. At the 3 year follow-up the patient was asymptomatic and reportedly satisfied with the surgery.

**Lessons:**

Traumatic AAD with an odontoid fracture is an exceedingly uncommon cervical spine injury. A lateral subluxation with a type II odontoid fracture in a patient with AS is rarer still, so much so that this type of subluxation was not classifiable using any of the previously developed classification systems. In this patient with AS, posterior OCF with internal fixation was necessary to avoid hardware failure, particularly in light of the intensive stress caused by AS.

## 1. Introduction

Although some reports concerning atlantoaxial subluxation/dislocation, rotatory fixation, and congenital atlantoaxial dislocation (AAD) have been published, documented instances of true traumatic AAD are rare. Rarer still are cases of traumatic AAD combined with odontoid fractures. What constitutes appropriate management in cases of traumatic AAD is still controversial due to the infrequency of this injury. Recently, we encountered a patient with traumatic lateral AAD, a type II odontoid fracture, and ankylosing spondylitis (AS). This case report has been approved by institutional review board of Jeonbuk National University Hospital, who waived the need for obtaining informed consent, because this study was retrospective.

## 2. Case report

**Figure 1.**
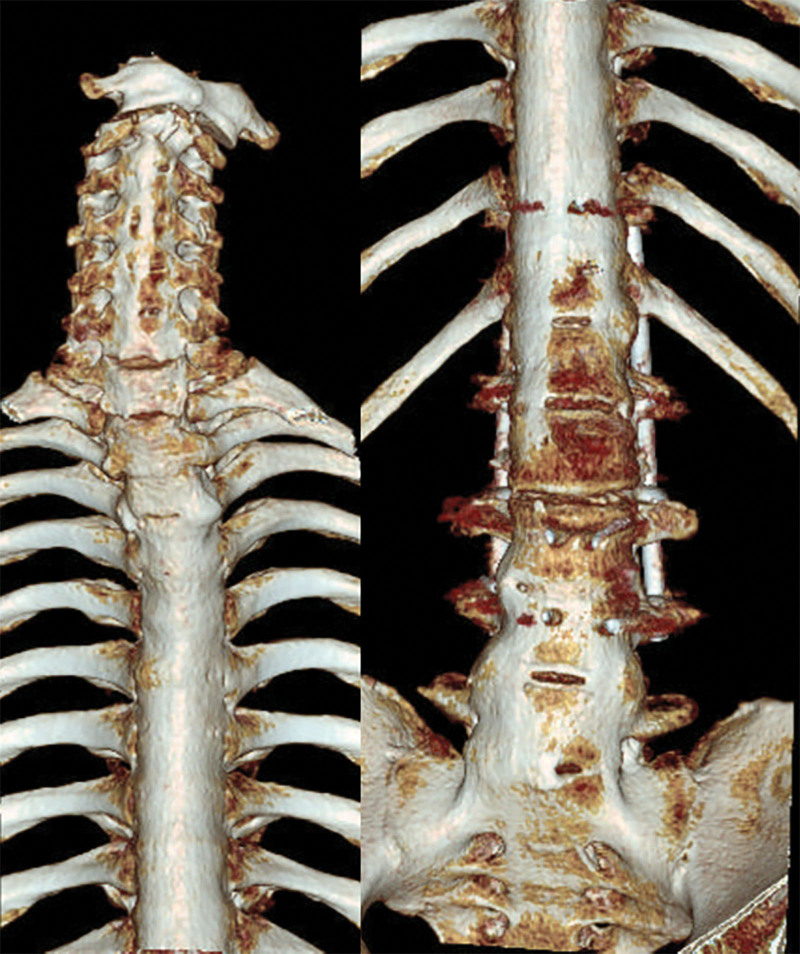
Preoperative spinal 3D computed tomography image showing ankylosis of the entire spine from the sacroiliac joint to the cervical spine.

**Figure 2.**
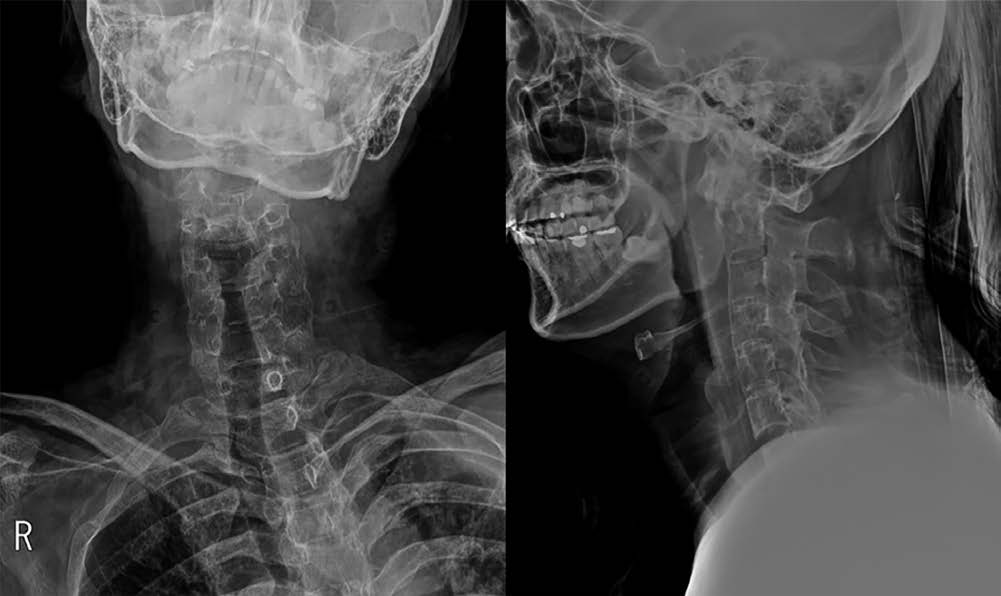
Preoperative X-ray showing that patient’s head was rotated to the right and there was an anterior subluxation of the C1 and odontoid tip relative to C2.

**Figure 3.**
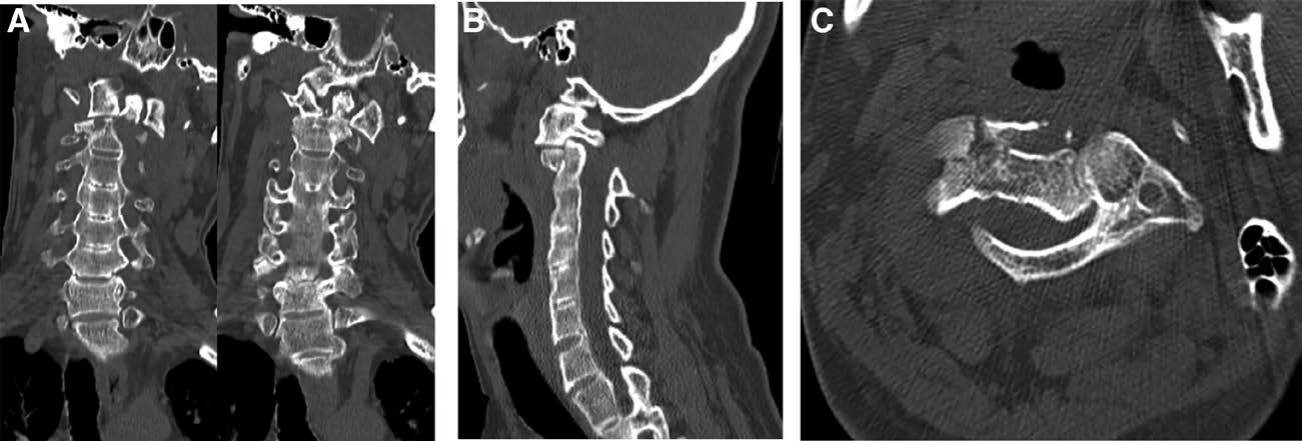
Preoperative coronal, sagittal and axial images. A coronal CT scan also revealed left lateral dislocation of C1 on C2 with a horizontal translation of the odontoid tip (A). On the axial and sagittal CT scan, the left C1 lateral mass was displaced anteriorly and locked by the C2 body (B and C). CT = computed tomography.

A 45-year-old male was sent to our emergency room after traffic accident. He presented with neck pain and stiffness, and with his head rotated to the right, but with no neurologic deficit. He had, >15 years previously, been diagnosed with AS. Spinal computed tomography (CT) showed ankylosis of the entire spine from the sacroiliac joint to the cervical spine (Fig. 1). On the cervical X-ray the patient’s head was rotated to the right and there was an anterior subluxation of the C1 and odontoid tip relative to C2 (Fig. 2). The coronal CT scan also revealed left lateral dislocation of C1 on C2 with a horizontal translation of the odontoid tip (Fig. 3A). On the axial and sagittal CT scan, the left C1 lateral mass was displaced anteriorly and locked by the C2 body (Fig. 3B and C).

Based on imaging, a diagnosis of lateral AAD (Fielding type III) combined with odontoid fracture (Anderson and D’Alonzo type II) was made.^[1,2]^ However, our case was unique in that the direction of AAD was true lateral.

**Figure 4.**
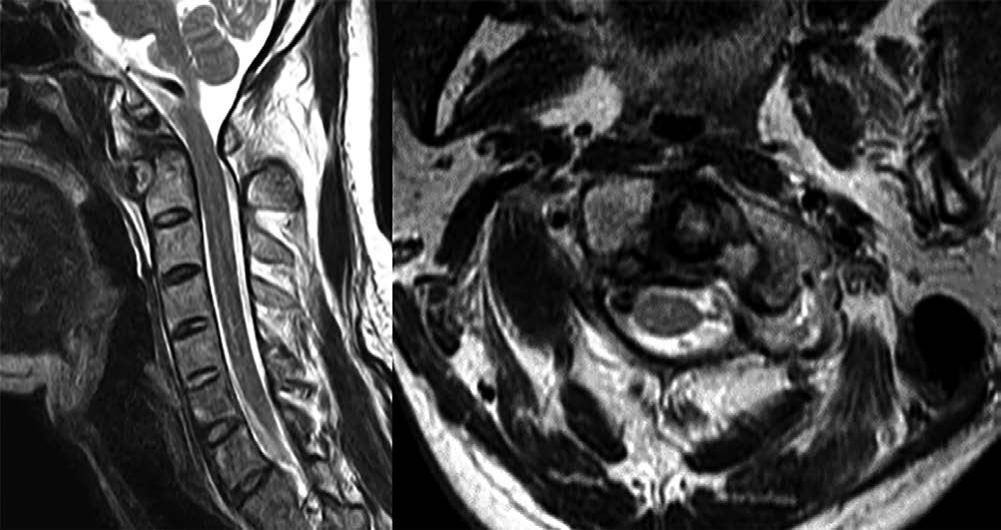
Preoperative cervical magnetic resonance image without contrast enhancement. Although the space available for the spinal cord was significantly reduced, there was no evidence that the spinal cord was compressed or injured.

Magnetic resonance imaging was performed to investigate the possibility of spinal cord injury. Although the space available for the spinal cord was significantly reduced, the spinal cord was not compressed and there was no evidence of spinal cord injury (Fig. 4).

**Figure 5.**
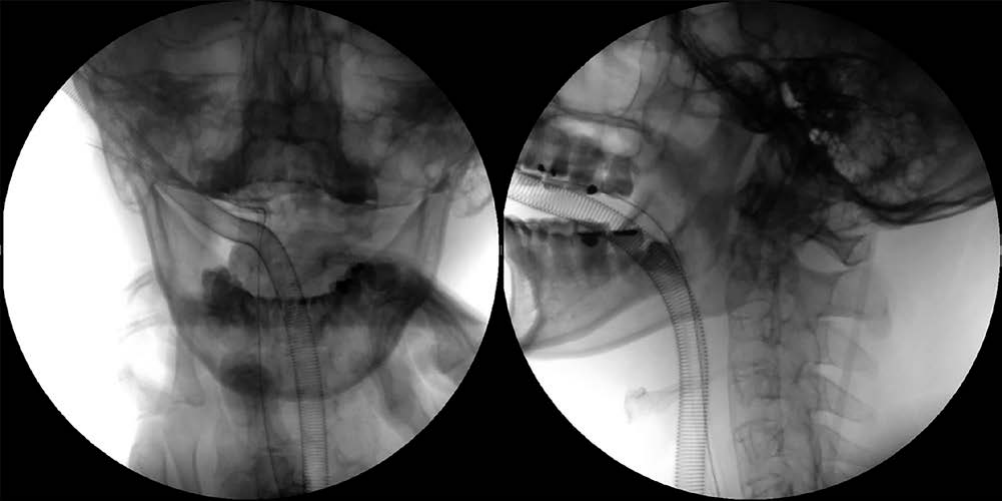
Intraoperative fluoroscopic images. Under general anesthesia, manual reduction was achieved and C1-C2 alignment was restored.

As the time after trauma was <7 hours, we attempted manual reduction. This failed as the patient complained of painful paresthesia in the left arm. Surgical treatment was scheduled for the following day. Under general anesthesia, we attempted a gentle manual reduction; this was fortunately achieved with a snapping sound. Reduction was confirmed by fluoroscopy (Fig. 5). The patient was then positioned prone on a frame with his head positioned in a Mayfield headrest system. The cervical spine was exposed in a standard fashion from the occiput to C5. Occipital plating and C2, C3, C4 screw fixation were performed, and a cancellous bone graft was added to the C1-C2 space.

**Figure 6.**
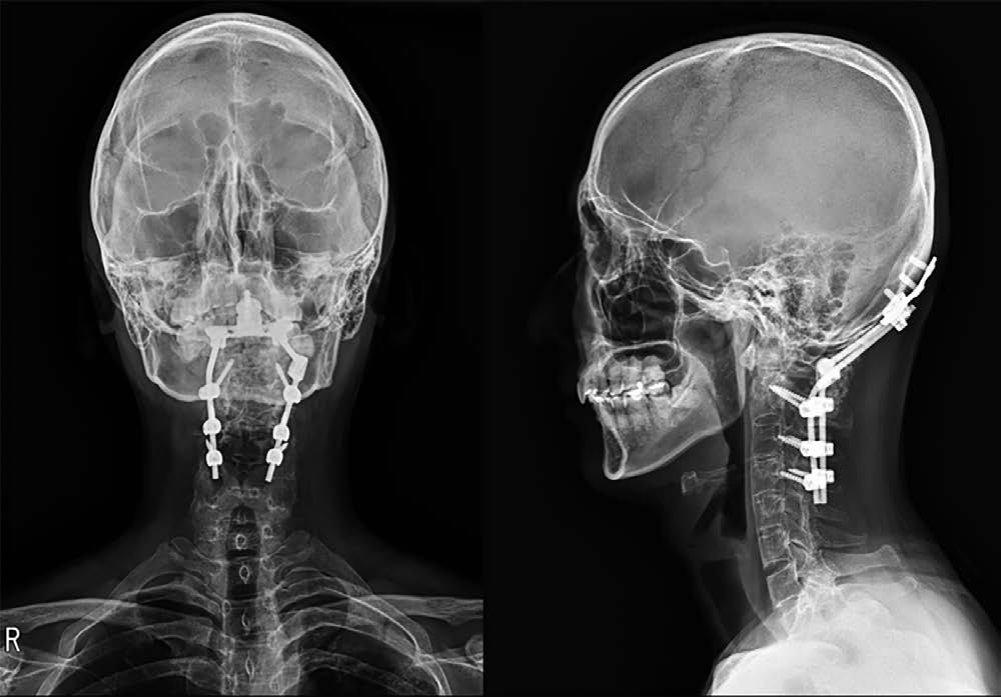
Post-operative X-ray showing a restoration of C1-C2 articulation and a reduction of the odontoid fracture.

The patient recovered from surgery without any complications. After surgery the patient was placed in a semirigid cervical collar for 8 weeks. The-post-operative X-ray & CT scans demonstrated a restoration of C1-C2 articulation and a reduction of the odontoid fracture (Fig. 6). At the 3 years follow-up, the patient was asymptomatic and satisfied with the surgery.

## 3. Discussion

### 3.1. Prevalence

Traumatic AAD is an often lethal and extremely uncommon injury. Traumatic AAD combined with type II odontoid fractures are even more rare, with only a few reports of such in the literature.^[3]^ Our case is different from even these reports, in as much as the AAD was true lateral. In fact, our literature review uncovered only two reports of traumatic lateral AAD combined with type II odontoid fracture.^[3,4]^ Lenehan et al presented a case of lateral C1-C2 dislocation complicating a type II odontoid fracture treated with open reduction, C1 lateral mass screws, and C2 pedicle screws.^[4]^ Musa et al also presented a case of traumatic atlantoaxial lateral subluxation with chronic type II odontoid fracture.^[3]^ Their case was treated by C1-C2 fusion with instrumentation and careful reduction of the dens. Our case is therefore unique; traumatic lateral AAD combined with a type II odontoid fracture. Moreover, our patient was diagnosed with AS over 15 years previously. As odontoid fractures are relatively rare in patients with AS, a gold standard treatment is lacking.^[5]^ To the best of our knowledge, a traumatic lateral AAD combined with a type II odontoid fracture in a patient with AS has not been reported before.

### 3.2. Treatment

Treatment of traumatic AAD aims to correct the sagittal alignment of the upper cervical spine, stabilize it to near anatomical alignment, and decompress the neural structure. There are no uniformly accepted treatment algorithms for AAD, with a great deal of variance in opinions concerning indications of nonoperative versus operative treatment modalities and which operative techniques are more appropriate. Some authors have highlighted the importance of individualizing treatment, particularly regarding the application of skeletal traction to effect reduction.^[6]^ In cases of traumatic AAD, reducing fracture dislocation is critical. In some cases, gentle closed manipulation with the use of an image intensifier may reduce the fracture dislocation. If manual reduction fails, skull traction (either administered at patient’s bedside or under general anesthesia and with fluoroscopic guidance) with gradual incremental increases in the applied traction weight, should be considered.^[7]^ After successful reduction, surgical treatment is necessary in most cases of traumatic AAD. A number of authors have recommended that transoral decompression combined with subsequent posterior fixation is the safest method of treatment for this condition.^[8,9]^ Goel and Laheri described procedures involving lateral mass screw instrumentation.^[10]^ Although both the transarticular and interarticular fixation methods are technically challenging and precise operations, they confer remarkable stability to the C1-C2 region. Transarticular fixation requires near-anatomical reduction prior to instrumentation. Alternatively, the Harms technique stabilizes the C1-C2 region by fixation with polyaxial screws and rods from the dorsal approach, effectively stabilizing the atlantoaxial complex.^[11]^

In light of the atlantoaxial instability, odontoid fracture, and the patient’s history of severe AS, we performed an occipito-cervical fusion (OCF). Post-surgery, the patient no longer complained of restricted neck movement. We assume that this was because the range of movement his neck was capable of had been reduced by AS prior to the traumatic injury.

### 3.3. The association with AS in traumatic AAD combined with odontoid fracture

Moderate-to-severe spinal restriction occurs during the late stage of AS in >50% of patients diagnosed with the disease, and it is usually complicated by vertebral osteoporosis because of prolonged immobilization.^[12]^ The rigidity and brittleness of the fused spine associated with AS increases the risk of fracture from minor traumas, as the spine’s altered biomechanics lead to it behaving more like an osteoporotic long bone rather than an elastic column.^[13,14]^ According to a systemic review by Westerveld et al, in approximately 65% of cases the precipitating trauma is low energy, and most fractures are cervical.^[15]^ In our case, as the patient had been previously diagnosed with AS over a decade ago, we suspect that he was already at high risk of atlantoaxial subluxation, and that the odontoid fracture and atlantoaxial subluxation were developed after only a minor trauma similar to insufficiency fractures. In short, AS contributed to the development of this unique fracture.

Physicians facing cases such as ours need to be take into consideration that a fracture in conjunction with a completely stiff spine may be particularly unstable, and that healing might be impaired by the long-term intake of nonsteroidal anti-inflammatory drugs and osteoporosis. Osteoporosis not only predisposes one to fractures but may also alter fracture healing.^[16]^ Accordingly, many authors recommend surgical treatment for such patients.^[17]^ Miao et al reported that they also performed OCF for odontoid fractures in patients with long-standing AS and that clinical outcomes were satisfactory.^[18]^ Considering our patient’s history of severe AS and the observed atlantoaxial instability in conjunction with the odontoid fracture, we likewise performed OCF to avoid hardware failure. Throughout the three years follow up process, the patient presented with no complaints and we observed no complications such as hardware failure or adjacent segment degeneration.

## 4. Conclusion

Traumatic AAD with an odontoid fracture is an exceedingly uncommon cervical spine injury. Lateral subluxation with a type II odontoid fracture in a patient with AS is rarer still, so much so that this type of subluxation was not classifiable according to any of the previously developed classification systems. Considering that the patient had been previously diagnosed with AS over 15 years ago, we suspect that he was at heightened risk of AAD and odontoid fracture, and that his AAD was the result of merely a minor trauma. In patients with AS, posterior OCF with internal fixation is necessary to avoid hardware failure, particularly in light of the intensive stress caused by the AS.

## Author contributions

**Conceptualization:** Youngmin Oh.

**Funding acquisition:** Youngmin Oh.

**Visualization:** Youngmin Oh.

**Writing – original draft:** Jongpil Eun, Youngmin Oh.

**Writing – review & editing:** Jongpil Eun, Youngmin Oh.
